# A cross-sectional historical study on the changes in self-esteem among Chinese adolescents from 1996 to 2019

**DOI:** 10.3389/fpsyg.2023.1280041

**Published:** 2023-12-01

**Authors:** Min Li, Qinghong Xu, Xiangwei Han, Yongzhi Jiang, Ru Ya, Jie Li

**Affiliations:** ^1^School of Psychology, Inner Mongolia Normal University, Hohhot, China; ^2^School of Education, Inner Mongolia University for Nationalities, Tongliao, China; ^3^Inner Mongolia Ethnic Education and Psychological Development Research Base, Tongliao, China; ^4^Inner Mongolia Autonomous Region Student Bullying Prevention and Control Research Center, Tongliao, China; ^5^School of Foreign Languages, Yulin University, Yulin, China; ^6^School of Education, Hulunbuir University, Hulunbuir, China

**Keywords:** self-esteem, SES, teens, cross-sectional historical studies, meta-analysis

## Abstract

This study aimed to investigate the changes in self-esteem levels among Chinese adolescents from 1996 to 2019. In this cross-sectional historical study, 109 articles using the Rosenberg Self-esteem Scale (SES) were selected from three Chinese and five English databases. The results showed that: (1) The self-esteem level of Chinese adolescents was positively correlated with the period, indicating that the self-esteem of Chinese adolescents was gradually increasing. (2) The increase in self-esteem level of girls was higher than that of boys. (3) The increase in the self-esteem level of only child was higher than that of non-only child. (4) The self-esteem level of rural adolescents increased year by year. However, the self-esteem level of urban adolescents was not significantly correlated with the years. (5) The changes in macro social factors can significantly predict the upward trend of the self-esteem level of Chinese adolescents.

## Introduction

Currently, China is experiencing economic and cultural development, social prosperity and stability, and the general improvement of residents’ living standards and quality of life. People are unsatisfied with low-level physiological and security needs but pursue higher self-esteem and self-realization needs. Bronfenbrenner’s ecosystem theory holds that the development of individual psychology is carried out in the interaction between the individual and the environment. Therefore, social progress and changes may lead to changes in the national self-esteem level. The adolescent stage is not only a critical period for the psychological and physical development of individuals but also a significant period for the cultivation of self-esteem (Liu and Xin, 2015). Self-esteem remains stable between the ages of 11 and 15 years and begins to grow significantly at the age of 15 (Harter, 1985; Orth and Robins, 2022). Children and adolescents shape their self-esteem through interactions with key influencers such as family, school, and peers (Chen et al., 2020; Veiga et al., 2021; Alcaide et al., 2023). Adolescents’ self-esteem is closely intertwined with their individual psychological development, as well as economic and social progression.

At the level of individual psychological development, self-esteem is recognized as a central factor in psychosocial adjustment (Orth and Robins, 2022). Self-esteem is not only closely related to interpersonal relationships (McFarland and Buehler, 1995) and academic achievement (Zhang et al., 2022). Self-esteem also has a buffering effect on anxiety (Brown et al., 1988). Individuals with high self-esteem tend to have better mental health (Twenge and Campbell, 2001). Individuals with low self-esteem are more likely to suffer from suicide, crime, anxiety, and depression (Trzesniewski et al., 2006; Wheeler, 2010; Sun, 2022).

At the economic and social development level, self-esteem is the cornerstone of social development. Festinger (1954) suggested that people tend to compare themselves with people who are doing better than them to Complete their tasks, i.e., upward social comparisons. High self-esteem individuals are more likely to choose upward social comparisons (Redersdorff and Martinot, 2003). Regarding behavioral outcome attributions, individuals with high self-esteem are more likely to reject and ignore the effects of failure and attribute failure to external factors (Hanaya, 2016). Also, self-esteem is closely related to academic achievement (Zhang et al., 2022). These upward social comparisons, rational attributions, and higher academic achievement are all factors that drive social development. Therefore, it is of great significance to conduct adolescent self-esteem research.

In the process of social change, the self-esteem of adolescents can be significantly influenced. Previous meta-analyses have indicated a notable decline in the scores of the Rosenberg Self-Esteem Scale among Chinese adolescents from 1996 to 2009 (Liu and Xin, 2015). This decline was accompanied by a gradual decrease in the mental well-being of Chinese secondary school students (Xin and Zhang, 2009). Sustained low self-esteem can lead to a range of internalizing problems such as depression, suicidal tendencies, eating disorders, and anxiety, as well as externalizing problems like violence and substance abuse (Mann et al., 2004). At the turn of the century, as society experienced changes and education rapidly developed, awareness of the importance of adolescent mental health deepened. The government issued several documents emphasizing the crucial role of mental health education and incorporated it into primary and secondary school curricula. Self-esteem serves as a fundamental characteristic of individual mental health and acts as a protective factor (Mann et al., 2004). The introduction of policies and educational support has epoch-making significance in improving adolescent self-esteem. Although meta-analyses have revealed changing trends in self-esteem among Chinese adolescents at the end of the 20th century, it remains crucial to examine whether there have been significant improvements in the self-esteem of China’s adolescent population under the influence of social change, policy emphasis, and educational development.

Moreover, gender differences have been observed in self-esteem levels. Males tend to exhibit higher self-esteem compared to females (Orth et al., 2018; Martinez-Escudero et al., 2023). However, females tend to score higher in academic or family self-concept (Reyes et al., 2023; Villarejo et al., 2023), while males score higher in emotional or physical self-concept (Chen et al., 2020; Alcaide et al., 2023). With this in mind, it is essential to understand the changing characteristics of self-esteem levels across different groups of Chinese adolescents over time. This study aims to address these questions by employing a meta-analytic approach to reveal the relationship between social change and changes in adolescents’ self-esteem in China over an extended period.

### Research status of self-esteem in Chinese adolescents

In China, some achievements have been made in describing the status quo and influencing factors of self-esteem. Still, there are few macro control and comprehensive analysis of adolescent self-esteem. For instance, the research conducted by Zhang et al., has demonstrated that adolescents exhibit low levels of self-esteem, which are further compounded by instability issues (Zhang et al., 2010). Moreover, self-esteem is positively associated with introversion and extroversion, while it is negatively correlated with anxiety and depression (Cai et al., 2006). Related research findings showed that reciprocal positive relationships between parental support and self-esteem (Yuan et al., 2023). low self-esteem was associated with decreased subjective well-being (Liu et al., 2023), and self-esteem partially mediated the link between emotional intelligence and creative self-efficacy (Chen and Cheng, 2023). It can be seen that Chinese scholars used to describe the self-esteem status of adolescents from the micro perspective, and the comprehensive analysis of adolescent self-esteem from the macro perspective can enrich the results of self-esteem research in China.

### A cross-sectional historical study

Cross-sectional historical research, or “meta-analysis of cross-sectional history,” differs from the general meta-analysis method. It mainly analyzes the chronological effect of psychological quantity over a long period through “post-doc validation.” This method was first proposed by Twenge (1997), who used it in a study of self-esteem among American college students. It confirmed that the self-esteem level of American college students would gradually increase over time (Twenge and Campbell, 2001). Chinese scholars Xin et al. first used this method to study psychological indicators such as mental health, loneliness, and self-confidence (Xin and Zhang, 2009; Xin and Xin, 2016, 2017). This method has also been used to explore the Marital Satisfaction of Chinese Couples, Subjective Well-Being of Chinese College Students (Su and Liu, 2020; Li et al., 2022). The results all showed that the change of years would have a specific impact on the psychological indicators of individuals. In addition, as a unique meta-analysis method, cross-sectional history research can analyze people’s psychological indicators and explore the impact of social changes on psychological indicators from the relationship between psychological indicators and social indicators.

### Social indicators

In cross-sectional history research, social indicators can explain part of the variation of individual psychological quantity (Twenge, 2000). Recent research by scholars at home and abroadhas utilized this approach to investigate the association between social indicators and adolescent self-esteem (Twenge and Campbell, 2001; Twenge et al., 2010b; Liu and Xin, 2015). The outcomes of these studies have revealed that adolescent self-esteem is related to various factors, such as divorce rates, unemployment rates, demographic shifts, and crime rates. Reducing the crime rate can help improve adolescents’ self-esteem. At the same time, the economy is significantly related to self-esteem and mental health (Twenge and Campbell, 2002; Yip, 2003; Leung and Xu, 2013). Specifically, the population is the main body of economic activity activities, and the aging rate of the population is accelerating, which means that adolescents will face greater social pressure and family pressure, which will impact adolescent self-esteem.

As a result, From the four perspectives of population characteristics, economy, education, and social threats, this study chooses the Gini coefficient, household consumption level (100 in 1978), *per capita* GDP index (100 in 1978), primary school enrollment rate, junior high school enrollment rate, senior high school enrollment rate, birth rate, death rate, natural population growth rate and juvenile crime rate To investigate the influence of these indices on the self-esteem of adolescents. It is worth mentioning that other researchers have also used these social indicators to measure the association between social indicators and psychological measures (Twenge and Jean, 2001; Twenge et al., 2010a; Shi et al., 2021).

In summary, this study will use a cross-sectional historical approach to explore the changes in adolescent self-esteem over time and reveal the changes in the mean self-esteem level of adolescents through a longitudinal perspective. At the same time, this study will reveal the influence of Chinese social and cultural background on adolescent self-esteem by examining the relationship between representative social indicators and adolescent self-esteem.

## Methods

### Research tool

The Rosenberg Self-esteem Scale (SES), established by Rosenberg in 1965, is a self-report scale designed to measure overall self-esteem, primarily among teenagers and college students (Twenge and Campbell, 2001). Consisting of 10 questions, the scale employs a four-point scoring system (1–4). The total score ranges from 10 to 40, with higher scores indicating higher levels of self-esteem and lower scores indicating lower levels of self-esteem. This scale has demonstrated good reliability and validity in domestic studies, with an internal consistency coefficient of 0.82 and a partial reliability of 0.81 (Tian et al., 2003). In terms of validity, research has indicated a significant correlation coefficient of 0.36 between the Coopersmith Scale (another self-esteem scale) and the FIS scale (self-esteem scale), as well as a correlation coefficient of 0.68 between the FIS scale and itself—both correlation coefficients being statistically significant (Cheng and Cui, 2010).

### Literature search

In this study, keywords, abstracts, and titles were based on the core terms of “adolescent self-esteem,” “Chinese adolescent SES,” and “Rosenberg Self-Esteem Scale,” The full text was searched in four Chinese databases, including China Knowledge, Wanfang, Wipro, and Excellent Master’s and Doctoral Dissertation Database. The core terms “adolescent self-esteem,” “Chinese adolescent SES” and “Rosenberg Self-Esteem Scale” were also searched in five English databases, including Web of Science, Ebsco, Ekspress and Rosenberg Self-Esteem Scale. Science, Ebsco, PsycArticles and Wiley, and a total of 597 articles were obtained.

### Inclusion criteria

The criteria for inclusion in the literature were: (1) the study was conducted on Chinese adolescents; (2) the sample size (N), mean (M), and standard deviation (SD) had to be reported completely in the literature; (3) the test had to be administered under normal conditions, not under adverse mood (e.g., anxiety) and special time (e.g., sleep time); (4) different articles published by the same author or authors were selected if they were taken from the same batch of data, then only the earliest article was selected.

**Figure 1:**
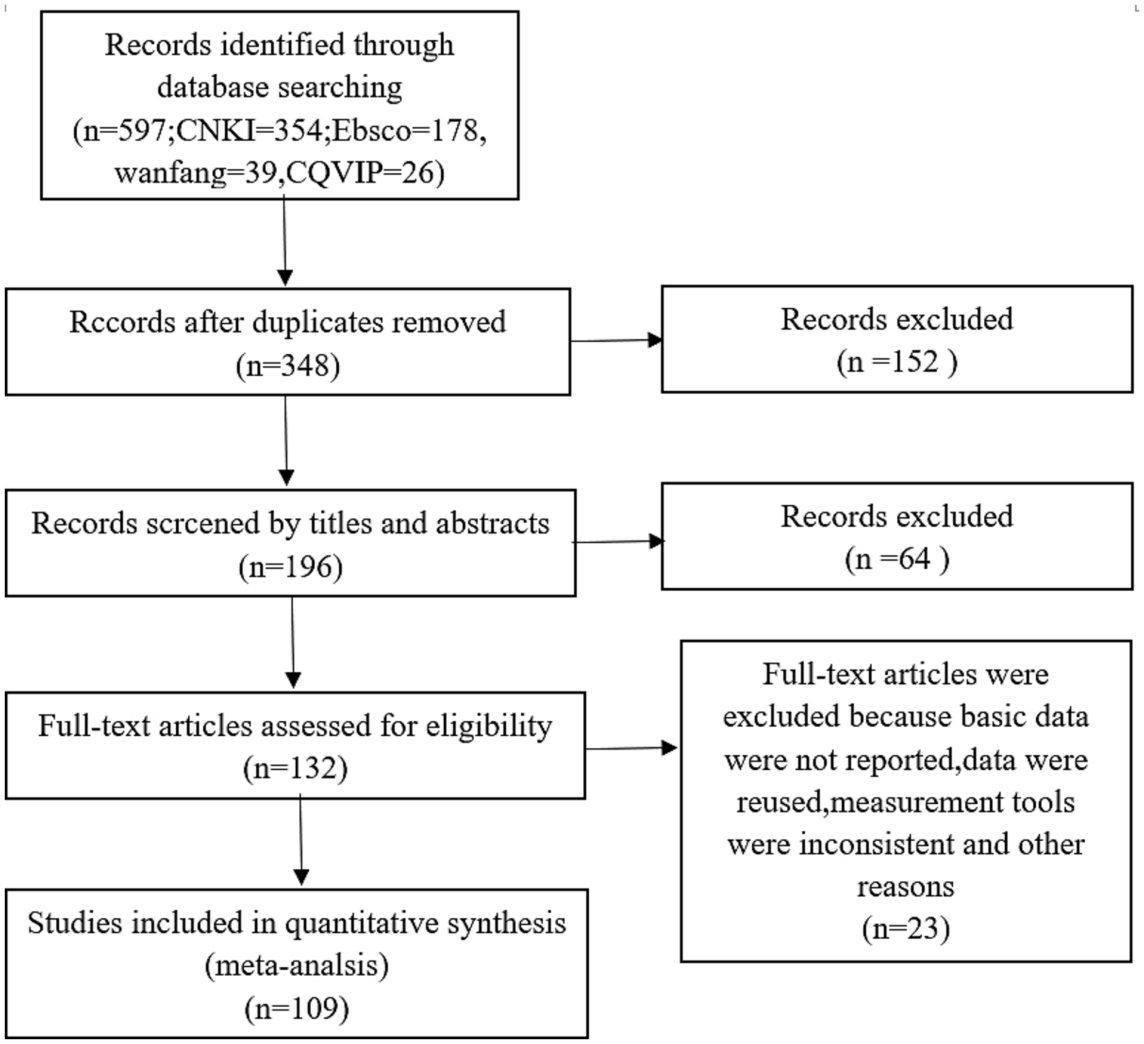
PRISMA flow chart for the screening of studies.

The exclusion criteria of the literature were: (1) due to the cultural differences between the East and the West, scholars in China are controversial about the eighth question of the SES scale, which is considered to have an impact on the results due to different interpretations, but if the eighth question is changed or deleted it will change the reliability and validity of the scale (Tian, 2006), therefore, the literature that changed the SES scale was deleted in this study, and (2) it was also deleted literature that used five-point scales, seven-point scales, etc. that were different from the general research. The PRISMA process for literature screening is shown in Figure 1.

### Literature coding and data collation

According to the above criteria, a total of 109 documents finally met the screening criteria of this study, including 104 documents in Chinese and 5 documents in English, with one or more documents in each year except 1997, 1998, 1999, and 2001.In previous studies, if the sampling year was not indicated in the documents, the data collection year was set as the year of publication minus 2 years (Xin and Zhang, 2009), so the chronology of this study is 1996–2019, totaling 24 years, involving a total sample of 115,140 people, including different subgroups, and the specific results are shown in Table 1.

**Table 1:** Adolescent self-esteem level of literature and distribution.

Year of sampling	Number of articles	Sample size	Gender		Urban and rural		The only child is not the only child	
			Male	Females	Towns	Rural	Only child	Non-only child
1996	1	190	0	0	0	0	0	0
2000	1	272	1	1	0	0	0	0
2002	3	1,146	0	0	0	1	0	0
2003	1	194	1	1	0	0	0	0
2004	2	1,617	2	2	0	0	0	0
2005	4	1,651	2	2	1	1	3	3
2006	5	2,198	1	2	0	0	1	1
2007	3	3,589	1	1	1	1	0	0
2008	3	1,162	3	3	1	1	0	0
2009	3	947	2	2	1	1	0	0
2010	8	26,810	4	4	0	0	2	2
2011	2	579	2	2	2	1	1	1
2012	10	7,129	6	6	3	3	2	2
2013	9	6,410	6	6	1	0	2	2
2014	14	12,758	5	5	3	3	3	3
2015	8	5,522	6	6	2	2	4	4
2016	5	4,268	2	2	2	1	2	2
2017	10	13,474	10	10	6	5	4	4
2018	9	4,970	6	6	4	4	2	2
2019	8	20,252	5	5	2	2	3	3
Total	109	115,140	65	66	26	26	29	29

The 109 pieces of literature deemed eligible were systematically categorized based on year of publication, gender (1 = Male, 2 = Female), place of origin (1 = Rural, 2 = Urban), only child or non-only child (1 = Only child, 2 = Non-only child), and region (0 = Undefined region, 1 = North China, 2 = South China, 3 = Northwest, 4 = Southwest, 5 = Northeast, 6 = Central China, 7 = East China, 8 = Multi-region). After that, the corresponding data, such as sample size, mean, standard deviation, and other relevant information, were appropriately documented in Table 2. To fully use the literature, the total sample data were entered into the database, and the sub-sample data were entered into the database. Although all the collected literature used four-point scoring, some used the 0–3 scoring method rather than the 1–4 method used in general scoring. The literature scored 0–3 using the mean plus 1 with constant standard deviation.

**Table 2:** Code assignment table for cross-sectional historical studies 1.

Coding	Number of documents	Sample size
Gender		
1 = Male	65	45,083
2 = Female	66	48,112
Place of origin		
1 = Rural	26	13,452
2 = Town	26	8,958
Only children versus non-only children		
1 = Only child	29	38,734
2 = Non-only child	29	29,699
Area		
0 = No definite area	13	122,523
1 = North China	12	104,617
2 = South China	7	41,579
3 = Northwest	8	78,338
4 = Southwest	12	92,504
5 = Northeast	5	43,985
6 = Central China	14	77,130
7 = East China	25	110,413
8 = multiple places	10	106,256
Total	109	115,140

### Sources of social indicators

This study has selected 10 social indicators: the Gini coefficient, household consumption level (measured as 100 in 1978), *per capita* GDP index (measured as 100 in 1978), primary school enrollment rate, junior high school enrollment rate, senior high school enrollment rate, birth rate, death rate, natural population growth rate and juvenile crime rate. These indicators were chosen based on their relevance to population characteristics, economy, education, and social threat. The data for these indicators were extracted from various sources, including the China Statistical Yearbook, China Household Survey Yearbook, and China Law Yearbook over a period of time. The selected indicators and their data are expected to provide comprehensive insights into China’s social development and trends.

### Data analysis strategy

General meta-analysis usually integrates the results of independent studies, analyzes them statistically, and ignores the trend of age changes. In contrast, cross-sectional historical research mainly explores the effects of age on psychological quantities over a considerable period. This study will analyze the differences between groups through the general meta-analysis method. At the same time, the cross-sectional history research method will be used to explore the relationship between the decade and the level of adolescent self-esteem through regression analysis and correlation analysis.

This study’s data analysis was divided into three parts. Firstly, we conducted a correlation analysis to explore the overall changes in adolescents’ self-esteem levels over the years. Secondly, to clarify the magnitude of changes in adolescents’ self-esteem levels over time, we calculated the effect size d and the chronological explanation rate r^2^ for the overall and each dimension. Finally, lagged correlation analysis was used based on previous studies to explore whether changes in social indicators could explain changes in adolescent self-esteem (Xin and Xin, 2017).

Specifically, this study will use the formula $d=M_{2019}-M_{1996}/SD$ to calculate the amount of effect, where d is the effect size, *M*_2019_ is the ending chronological age, *M*_1996_ is the starting chronological age, and SD is the mean and standard deviation of all studies. The mean scores of self-esteem levels of adolescents in the starting and ending years were obtained through regression equations. We took age as the independent variable and the self-esteem scores obtained after weighting according to sample size as the dependent variable and established a regression equation: y = Bx + C. Here, y represents the weighted self-esteem score, B represents the standardized regression coefficient, x represents the chronological age, and C represents the constant term. Establishing the regression equation allowed predicting the mean self-esteem levels *M*_1996_ and *M*_2019_ in 1996 and 2019. sd was obtained by calculating the mean, and standard deviation across all studies, a method that may avoid the ecological fallacy (Rosenthal et al., 2000). The chronological interpretation rate r^2^ will also be calculated as r^2^ = d^2^/(d^2^ + 4) to make the results more accurate.

The social indicators for each era were matched to the mean self-esteem scores of each study separately, with two types of matching, one for the year of data collection and the other for the 5 years prior to data collection. For example, the self-esteem scores of each study in 2019 were matched with each social indicator in 2014 and 2019.Twenger argues that if social indicators can explain the changes in Chinese adolescents’ self-esteem levels over the eras, then the correlation between the adolescent self-esteem scores and the social indicators in the previous 5 years should be significant when the adolescents’ self-esteem scores are matched with the social indicators in the previous 5 years. In addition, if there is a link between social indicators and adolescent self-esteem levels, then the relationship between the two should also be significantly correlated in the year of data collection.

## Findings

### Overall changes in adolescent self-esteem levels over time

**Figure 2:**
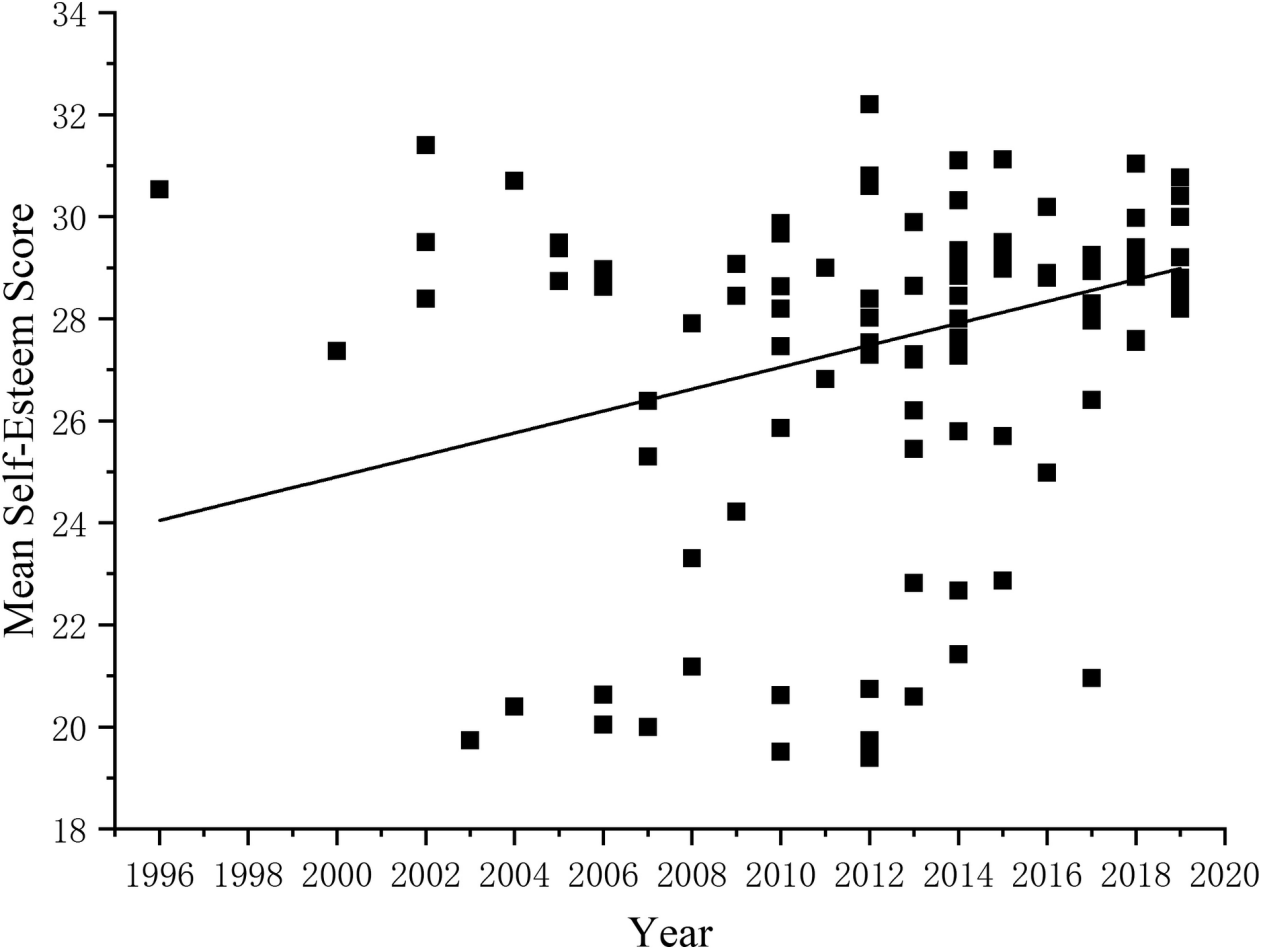
The change in self-esteem level of adolescents from 1996 to 2019.

A total of 109 articles covering 24 years and 115,140 adolescents were identified. Figure 2 directly illustrates the changing trend of the self-esteem level of adolescents over time since 1996. The FIGURE shows adolescents’ self-esteem level changes with time as the abscissa and the mean self-esteem score as the ordinate. The results showed that the self-esteem level of adolescents gradually increased over time from 1996 to 2019. In addition, the linear fitting results also proved that the linear model could better fit the relationship between age and self-esteem (*F* = 6.26, *p* < 0.05).

To more accurately quantify and describe the changes in the self-esteem level of adolescents, a correlation analysis was conducted between the period and the mean self-esteem score according to the method adopted by previous researchers (Xin et al., 2012). The results showed that the self-esteem level of Chinese adolescents was significantly positively correlated with the period (r = 0.24, p < 0.05). This indicates that from 1996 to 2019, the self-esteem level of Chinese adolescents shows an upward trend.

### The changes and explanatory rate of adolescent self-esteem level over the years

Although the above content shows that the adolescent self-esteem level changes with the years but cannot specifically describe the magnitude of the change, to explore the magnitude of changes in adolescent self-esteem over 24 years, we used the methodology of previous studies (Twenge and Campbell, 2001; Xin and Zhang, 2011). This study will use the effect size d and the explanatory rate r^2^ to illustrate the changes in adolescent self-esteem. First, with age as the independent variable and the weighted self-esteem score as the dependent variable, a regression equation was established: y = 0.23X−443.57 (where y is the weighted average self-esteem score, 0.23 is the standard regression coefficient，and x is the year of data collection). Then, by introducing the starting and ending years into the linear regression equation, the average self-esteem score M in 1996 and 2019 can be obtained_1996_ = 15.51, *M*_2019_ = 20.80. Calculate *M* again_1996_ And *M*_2019_The difference, divided by the standard deviation of the mean, SD, gives the effect size d and the explanatory rate r by decade^2^.

The results showed that from 1996 to 2019, adolescents’ self-esteem scores increased by 5.29 points, with an effect size of 1.15 (d = 1.15). According to Cohen’s categorization, an absolute value of the effect size greater than 0.8 is considered a large effect, an absolute value between 0.5 and 0.8 suggests a medium effect, and a range of 0.2 to 0.5 indicates small effects (Cohen and Jacob, 1992). Applying Cohen’s definition, a significant increase in self-esteem would be considered a large effect. It is worth noting that the level of self-esteem among Chinese adolescents has shown a substantial increase from 1996 to 2019, with the chronological factor accounting for 25% of the variance in self-esteem scores (r^2^ = 0.25).

### Mean levels of adolescent self-esteem as a function of grade level

**Figure 3:**
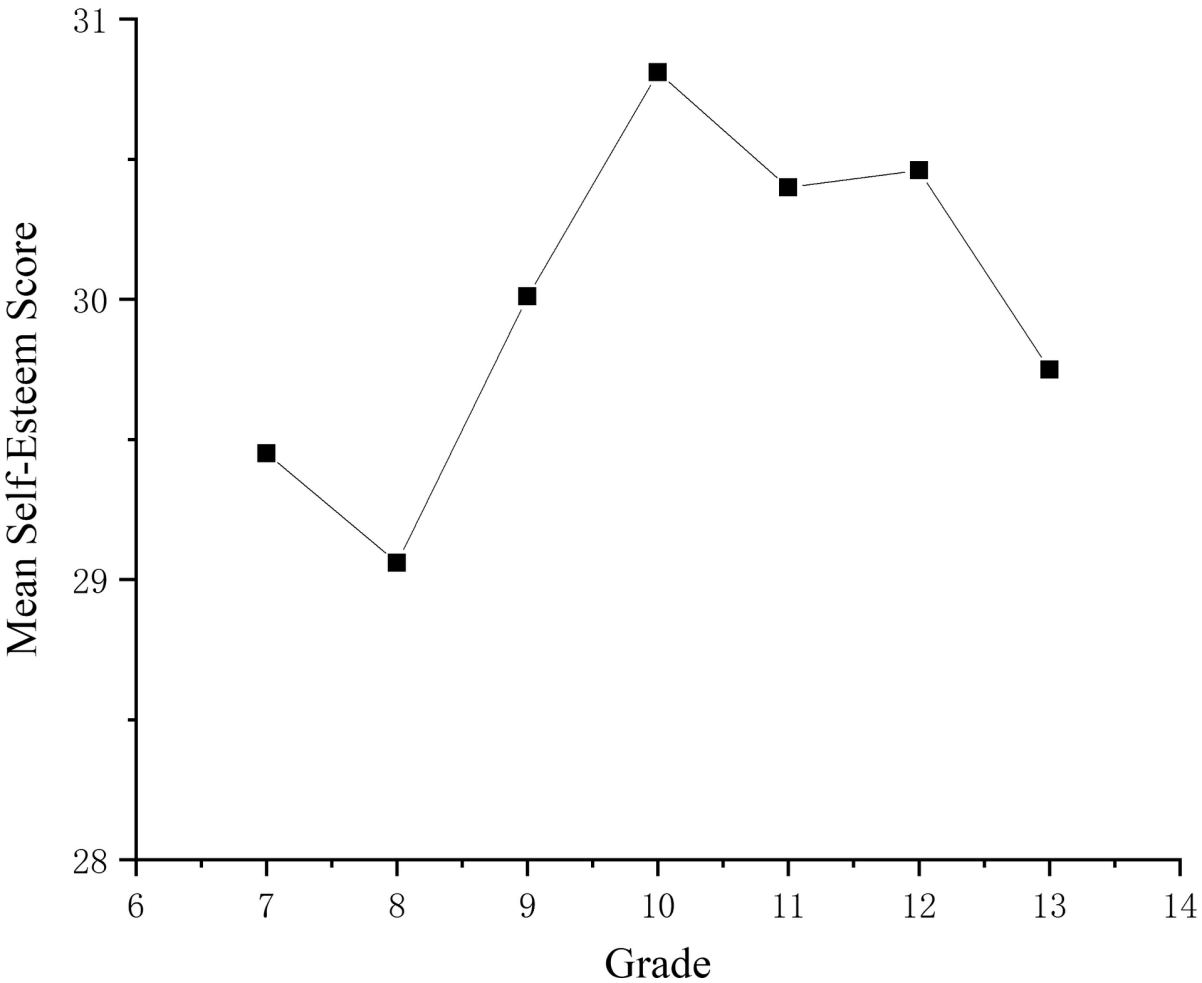
Differences in self-esteem levels by grade level. The age mean was obtained by fixing the covariates at the following values: era = 2006.75, region = 4.11.

How does self-esteem develop among Chinese adolescents? To answer this question, a one-way analysis of variance (ANOVA) was conducted with grade level and self-esteem score means as independent and dependent variables, chronological age and region as covariates, and sample size as a weight, which resulted in corrected means for each grade level (Figure 3). The results showed that the overall level of self-esteem increased with age. The mean of self-esteem for students in grade 7 (first year) was 29.45. the mean of self-esteem for students in grade 13 (first year of college) became 29.75. the overall level of self-esteem increased by 0.07 standard deviations (d = 0.07), and further analyses revealed that in the three-year period between grades 8 and 10 the overall level of self-esteem increased by 1.75, 0.41 standard deviations. According to Cohen’s definition (1977), this is a small effect (0.2 < d < 0.5). Although self-esteem levels among adolescents generally trended upward, self-esteem scores declined in grades 7 (freshman year), 10 (senior year), and 12 (senior year), with the most significant declines occurring in high school from grades 12 through 13, with a decline of 0.71 points and 0.18 standard deviations.

### Gender differences in adolescent self-esteem

**Figure 4:**
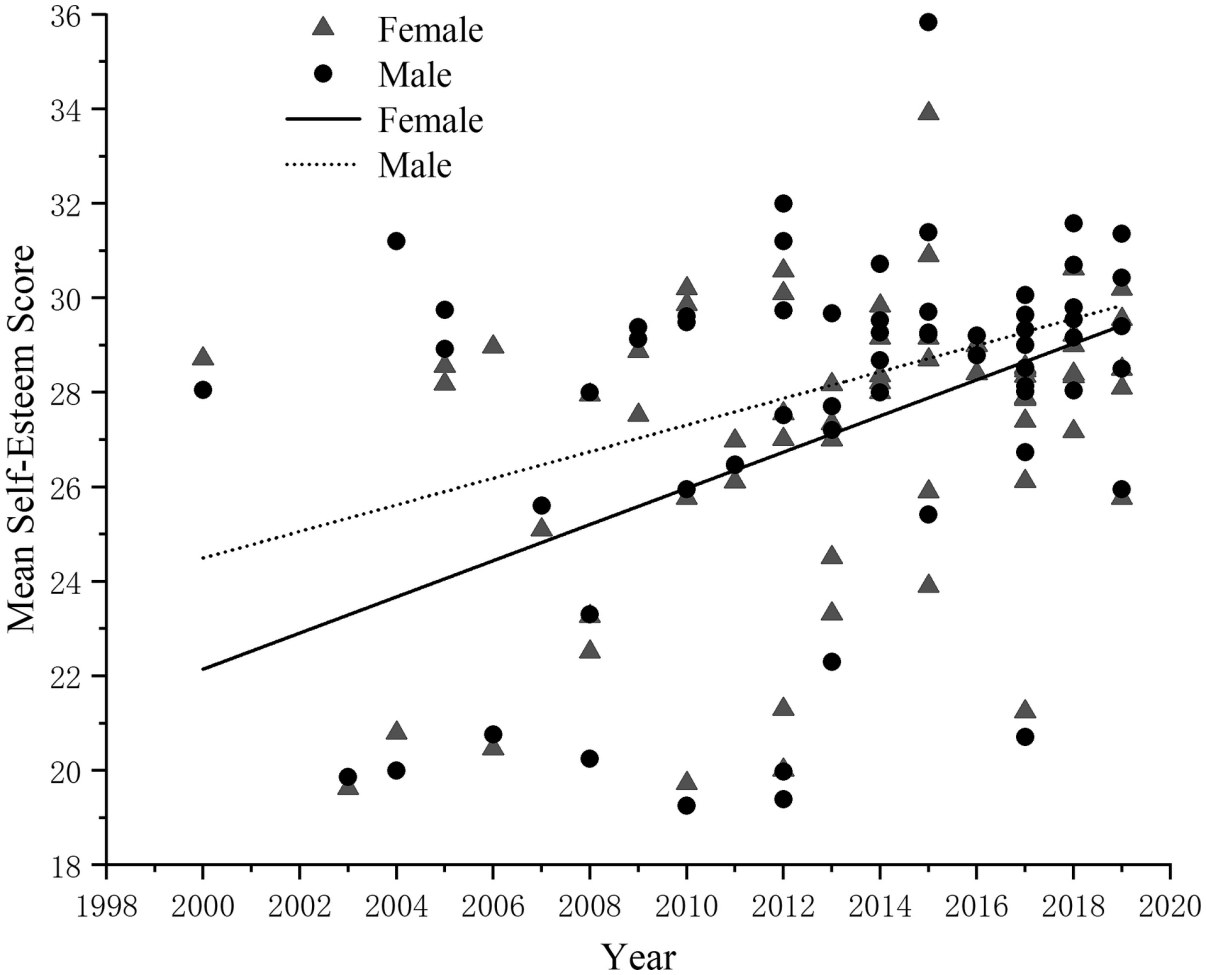
The change in boys’ self-esteem levels over the years.

In order to compare the level of self-esteem among adolescents of different genders, a total of 66 papers on gender differences were collected for this paper. Among the 66 articles, a total of 93,195 samples were included, consisting of 45,083 boys (48.37%) and 48,112 girls (51.62%). There was at least one piece of literature available for each year, except for the years prior to 2000 and 2001. In order to explore the trend of self-esteem level of adolescents of different genders, scatter plots of self-esteem scores of boys and girls were established (Figure 4) and correlation analysis was conducted. It was found that the self-esteem levels of both boys (r = 0.28, *p* < 0. 05) and girls (r = 0.34, *p* < 0. 01) showed an increasing trend over the years.

The same data analysis method was used to conduct a cross-sectional historical meta-analysis on boys and girls. The decade could significantly predict the change in the self-esteem level of boys and girls, and the self-esteem level of boys and girls gradually increased with the years. From 2000 to 2019, boys’ self-esteem scores increased by 5.32 points, the effect size is 1.14 (d = 1.14), and the decade explained 25% of the variation in boys’ self-esteem scores [(r = 0.97, *p* < 0.01)^2^ = 0.25]; Girls’ self-esteem scores increased by 7.22 points and the effect size is 1.60 (d = 1.60), and decade explained 39% of the variation in girls’ self-esteem scores (r^2^ = 0.39). According to Cohen’s definition, the effect sizes for both boys and girls over the years were large, but the increase in self-esteem levels was greater for girls than for boys. This shows that the level of self-esteem of adolescents of different genders has increased over the years, while the increase in the level of self-esteem of girls is greater than that of boys.

This study used a general meta-analysis with male students as the experimental group and female students as the control group to test gender differences in adolescents’ self-esteem levels through the formula. The gender difference effect size for each study was calculated using the formula $d=M_{e}-M_{c}/SD$ , where *d* is the effect size. Female students were used as the experimental group *M*_*e*,_ and male students as the control group *M*_*c*,_ *SD* is the joint standard deviation of the two groups. In the formula $SD=\sqrt{\left[\left(n_{e}-1\right)S_{e}^{2}+\left(n_{c}-1\right)S_{c}^{2}\right]/(n_{e}+n_{c}-2}$ , where n_e_ and *S_e_*^2^ are the sample size and variance of female students in each study, *n*_c_ and *S_c_*^2^ are the sample size and variance of male students in each study, respectively. Then, the weights of each study were calculated W_i_, $W_{i}=2N_{i}/\left(8+d_{i}^{2}\right)$ , Where *N_i_* is the sample size of each study. Finally, the mean effect size of the difference between the means of the two groups was calculated according to Equation $\bar{d}=\sum w_{i}d_{i}/\sum w_{i}$ .

Where d is the effect size, W_i_ is the weight of each study, *N_i_* is the sample size of each study, *M_e_* and *M_c_* are the mean self-esteem scores of the experimental and control groups respectively, and SD is the joint standard deviation of the two groups. The mean effect size calculated was 0.26, and Cohen considered 0.26 as a small effect, indicating a small gender difference in self-esteem.

The changes and differences in self-esteem between only child and non-only child.

**Figure 5:**
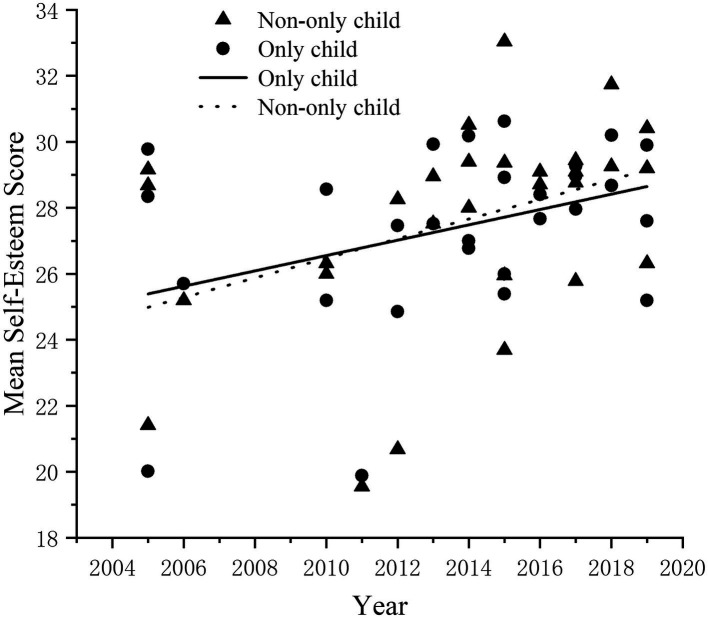
The change in self-esteem level of only child over time.

In the present study, a total of 29 papers reported the results of sub-studies focusing on only children and non-only children separately. The combined sample size for these sub-studies was 68,433, with 38,734 (56.60%) being only children and 29,699 (43.40%) being non-only children. The data were collected from 2005 to 2019. To explore the changing trend of the self-esteem level of only child and non-only child, scatter plots of the self-esteem scores of only child and non-only child were established (Figure 5), and the correlation analysis was conducted. The results showed that the self-esteem level of only child (r = 0.41, *p* < 0.05) and non-only child (r = 0.37, *p* < 0.05) showed a gradually increasing trend.

A cross-sectional meta-analysis was conducted on only child and non-only child, and the results showed that the self-esteem score of only child increased by 4.20 points and the effect size is 0.91(d = 0.91). According to Cohen’s definition, the effect size for the chronology variable was large, explaining 17% of the variance in the self-esteem scores of only children (r^2^ = 0.17). Specifically, the self-esteem scores of children with siblings increased by 3.22 points, resulting in an effect size of 0.74 (d = 0.74). In the case of non-only children, the effect size for the chronology variable was medium, explaining 12% of the variance in their self-esteem scores (r^2^ = 0.12). The results showed that the self-esteem level of only child and non-only child increased significantly, and the self-esteem of only child was higher than that of the non-only child.

In order to explore whether there is a difference in the level of self-esteem between only children and non-only children, the same method of gender difference test was used to analyze the results. It was found that the mean effect size, d = 0.8, which is a large effect size according to Cohen’s definition, indicates a significant difference in self-esteem levels between only children and non-only children.

### Changes in self-esteem levels between urban and rural adolescents over the years

**Figure 6:**
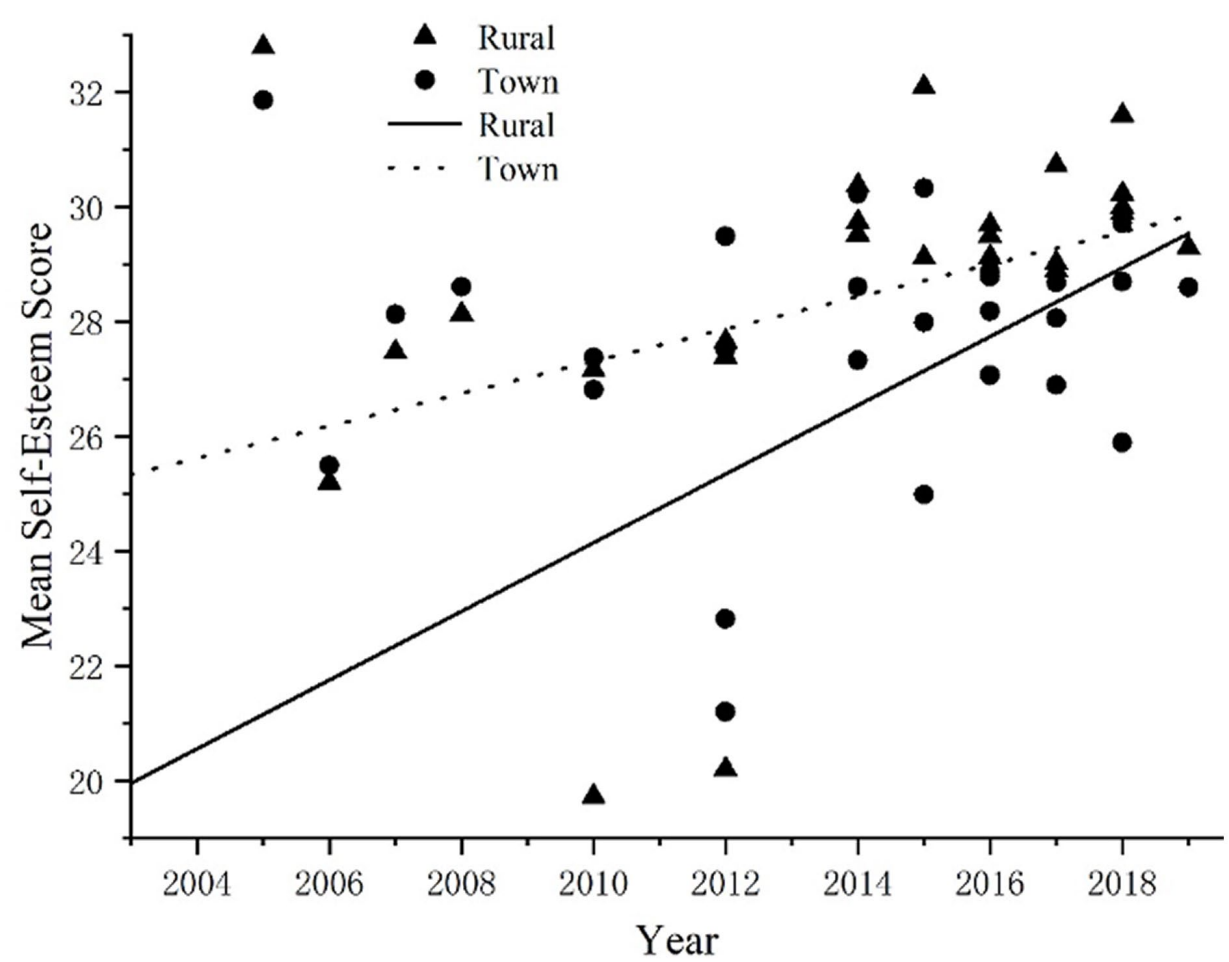
Changes in self-esteem levels over time among rural adolescents.

In this study, a total of 26 articles reported the sub-research results regarding the place of birth. The combined sample size for these studies was 22,410 persons, with 13,452 (60.02%) being from rural areas and 8,958 (39.98%) from urban areas. The samples used in these studies were collected between the years 2002 and 2019. Results of correlation analysis showed that the self-esteem level of rural adolescents was significantly correlated with the years (r = 0.45, *p* < 0.05), while the self-esteem level of urban adolescents was not significantly correlated with the years. That is, the years could significantly predict the change in the self-esteem level of rural adolescents but could not predict the changing trend of the self-esteem level of urban adolescents. According to the scatter plot (Figure 6), the self-esteem level of rural adolescents showed an upward trend. The self-esteem scores of rural adolescents increased by 6.12 points and the effect size is 1.5 (d = 1.50). According to Cohen’s definition, the effect size for the chronology variable was large, explaining 36% of the variance in the self-esteem scores of rural adolescents (r^2^ = 0.36).

To investigate the potential differences in self-esteem levels between rural and urban adolescents, the same gender difference testing method was employed for analysis. The results revealed a mean effect size of d = 0.13, suggesting that there is no significant difference in self-esteem levels between rural and urban adolescents.

### Correlation between adolescent self-esteem and social indicators

The above results show adolescent self-esteem levels over the years, so how does this change relate to social changes? As previously mentioned, cross-sectional historical research can be utilized to investigate the impact of social factors on psychological quantity by closely analyzing the correlation between social indicators and the scores of a specific psychological quantity within a certain group. The results of the correlation analysis between social indicators and self-esteem scores of adolescents in the current year are shown in Table 3. The self-esteem of adolescents as a whole and its subgroups is significantly positively correlated with the level of household consumption, *per capita* GDP, junior high school entrance rate, and population mortality, and negatively correlated with the crime rate of adolescents. This indicated that residents’ consumption level, GDP *per capita*, junior high school enrollment rate, population mortality rate, and juvenile crime rate significantly influenced adolescent self-esteem.

**Table 3:** Correlation between social indicators and mean self-esteem levels in the current year.

Social indicators	Overall	Boys	Girls	Only child	Non-only child	Town	Rural
Gini coefficient	0.37	0.24	0.23	0.61^*^	0.55	0.98^**^	0.85^**^
Household consumption level	0.55^*^	0.50^*^	0.50^*^	0.60^*^	0.58^*^	0.59	0.39
Index of gross domestic product *per capita*	0.56^*^	0.51^*^	0.50^*^	0.59^*^	0.57^*^	0.61	0.41
Primary school enrollment rate	0.32	0.26	0.03	0.55	0.36	0.97^**^	0.51
Junior high school enrollment rate	0.52^*^	0.46	0.50^*^	0.50	0.44	0.75	0.58^*^
High school admission rate	0.29	0.40	0.21	0.61	0.52	1.0^**^	0.03
Birth rate	0.28	0.18	0.09	0.04	0.18	0.93^*^	0.24
mortality rate	0.38	0.38	0.50^*^	0.16	0.12	0.46	0.47
Natural population growth rate	0.34	0.08	0.03	0.15	0.21	0.91^*^	0.33
Juvenile crime rate	0.41	0.52^*^	0.43	0.64^*^	0.56^*^	0.60	0.22

^*^p < 0.05, ^**^p < 0.01.

This study uses the lag correlation analysis method to investigate the nexus between social environment and adolescent self-esteem (Xin and Xin, 2017). In particular, Data on social indicators from the previous 5 years and the following 5 years were collected and correlated with the self-esteem scores of the current year. If the social indicators from 5 years ago are significantly correlated with the self-esteem scores of that year, it means that the social indicators from 5 years ago are important factors influencing the development of adolescents’ self-esteem, and if the social indicators from 5 years later are significantly correlated with the self-esteem scores of that year, it means that these social indicators are able to predict the trend of the future changes in adolescents’ self-esteem. However, due to time constraints, the data on social indicators can only be collected until 2020, so the lagged correlation analysis after 5 years actually seeks the correlation between the social indicators between 2001 and 2020 and the average of self-esteem water between 1996 and 2019. The results show that the social indicators 5 years ago (Table 4), except for the high school advancement rate, are all significantly correlated with adolescents as a whole or their subgroups, i.e., the social indicators 5 years ago will have a certain impact on the self-esteem of adolescents 5 years later, which suggests that the social indicators 5 years ago are an important factor influencing the level of adolescents’ self-esteem.

**Table 4:** Correlation between social indicators and mean self-esteem level 5 years ago.

Social indicators	Overall	Boys	Girls	Only child	Non-only child	Town	Rural
Gini coefficient	0.43	0.31	0.50^*^	0.12	0.01	0.90^*^	0.40
Household consumption level	0.56^*^	0.48^*^	0.48^*^	0.59^*^	0.59^*^	0.52	0.38
Index of gross domestic product *per capita*	0.55^*^	0.50^*^	0.49^*^	0.60^*^	0.59^*^	0.55	0.37
Primary school enrollment rate	0.46^*^	0.35	0.30	0.30	0.21	0.67	0.41
Junior high school enrollment rate	0.55^*^	0.56^*^	0.52^*^	0.60^*^	0.54	0.72	0.33
High school enrollment rate	0.22	0.12	0.17	0.14	0.30	0.13	0.27
Birth rate	0.45^*^	0.27	0.28	0.10	0.19	0.71	0.57^*^
mortality rate	0.46^*^	0.57^*^	0.50^*^	0.64^*^	0.56^*^	0.60	0.19
Natural population growth rate	0.50^*^	0.37	0.36	0.11	0.01	0.73	0.58^*^
Juvenile crime rate	0.19	0.21	0.22	0.49	0.56^*^	0.46	0.47

^*^p < 0.05, ^**^p < 0.01.

The Gini coefficient, the level of consumption, GDP *per capita*, the rate of transition to junior high school, the population mortality rate, and the juvenile delinquency rate were all significantly correlated with juveniles as a whole or with their subgroups at the end of the five-year period (Table 5). This suggests that the Gini coefficient, the level of consumption of the population, GDP *per capita*, the rate of advancement to junior high school, the population mortality rate, and the juvenile delinquency rate are all predictive of the level of self-esteem of adolescents. In summary, these social indicators may have influenced the development of self-esteem among Chinese adolescents.

**Table 5:** Correlation between social indicators and the mean value of self-esteem water after 5 years.

Social indicators	Overall	Boys	Girls	Only child	Non-only child	Town	Rural
Gini coefficient	0.40	−0.48^*^	−0.27	−0.61^*^	−0.55	−0.26	0.85^**^
Household consumption level	0.60^**^	0.57^*^	0.60^*^	0.60^*^	0.58^*^	0.27	0.39
Index of gross domestic product *per capita*	0.60^**^	0.59^*^	0.62^**^	0.59^*^	0.57^*^	0.23	0.41
Primary school enrollment rate	0.24	−0.30	0.20	−0.55	−0.36	−0.22	0.51
Junior high school enrollment rate	0.63^*^	0.64^**^	0.75^**^	0.50	0.44	0.05	0.58^*^
High school enrollment rate	0.40	0.48	0.29	0.61	0.52	0.50	−0.03
Birth rate	−0.06	0.16	0.04	0.04	−0.19	−0.40	−0.24
mortality rate	0.44	0.48	0.64^**^	0.16	0.11	−0.41	0.47
Natural population growth rate	−0.17	0.04	−0.13	0.15	−0.21	−0.35	−0.33
Juvenile crime rate	−0.51^*^	−0.57^*^	−0.51^*^	−0.65^*^	−0.56^*^	−0.25	−0.22

^*^p < 0.05, ^**^p < 0.01.

## Discussion

### The overall level of adolescent self-esteem is gradually increasing

This study conducted a cross-sectional historical study on 109 pieces of literature using Rosenberg Self-esteem Scale between 1996 and 2019. The results showed that the mean self-esteem scores of Chinese adolescents were significantly positively correlated with the period, and the mean self-esteem scores increased by 1.15 standard deviations. This indicates that the self-esteem level of Chinese adolescents has gradually increased with age. This change in age reflects the impact of the macro social background on the self-esteem of adolescents, which is consistent with the conclusions of many Chinese scholars (Liu and Zou, 2007; Li et al., 2008).

**Figure 7:**
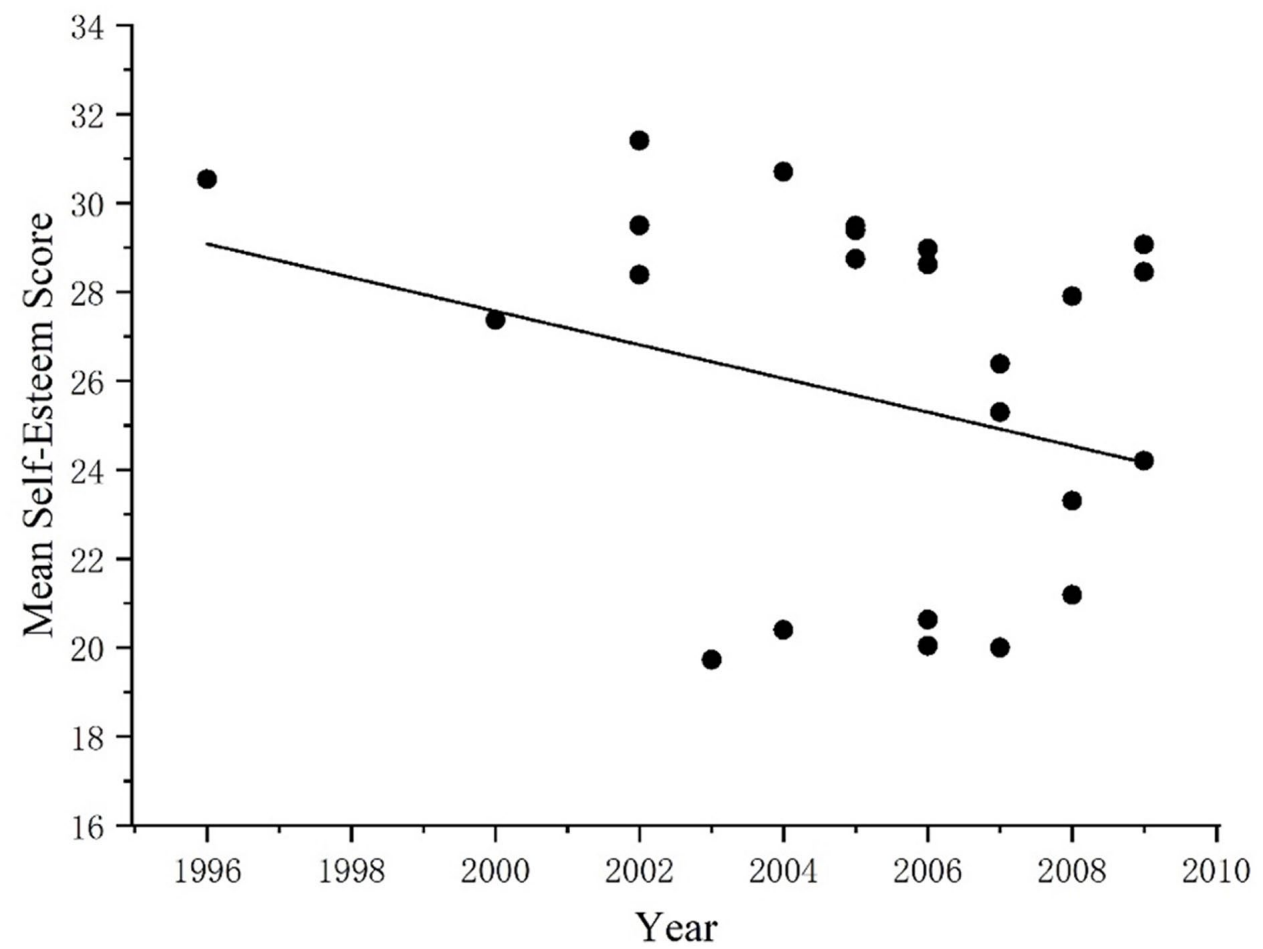
Changes in adolescent self-esteem levels from 1996 to 2009.

**Figure 8:**
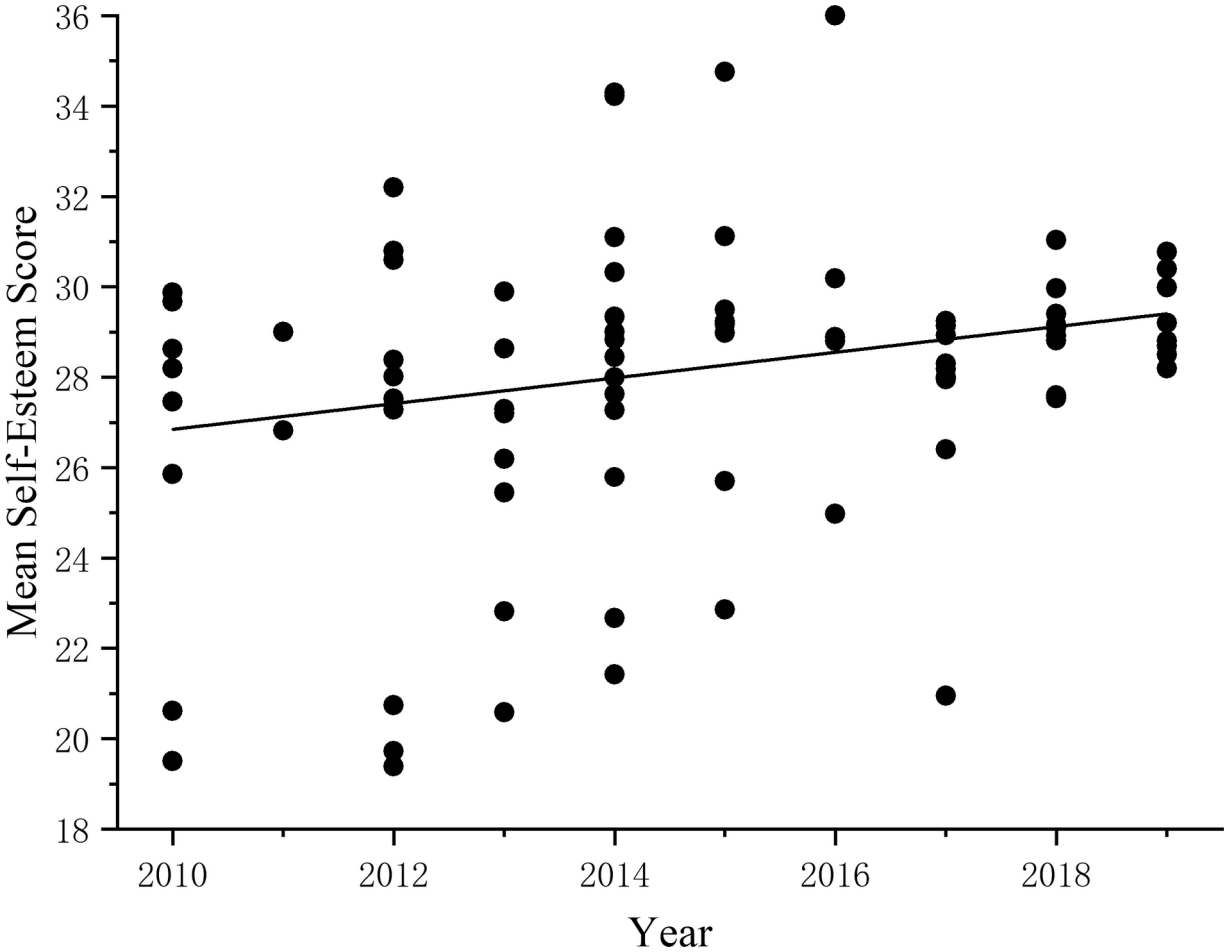
Changes in adolescent self-esteem levels from 2010 to 2019.

In addition, Liu et al. conducted a cross-sectional historical meta-analysis of the literature using SES to measure Chinese adolescents from 1996 to 2009 (Liu and Xin, 2015), and the results showed that the self-esteem scores of adolescents decreased over time, which was inconsistent with the conclusions of this study. The comparative analysis found that this study covered 10 years more than Liu et al.’s study. Scatter plots of adolescents’ self-esteem scores from 1996 to 2009 and from 2010 to 2019 were established, respectively (Figures 7, 8), to further clarify the change in the self-esteem level of Chinese adolescents from 2009 to 2019. The results show that the changing trend of self-esteem scores from 1996 to 2009 is consistent with the conclusion of Xin Ziqiang et al. Chinese adolescents’ self-esteem level shows a downward trend, but since 2010, the self-esteem scores of adolescents have gradually increased. These results reveal historical differences in self-esteem over a historical period, which is consistent with other studies that have also found intergenerational differences, such as in parenting practices (Chen et al., 2020).

There may be several reasons for improving self-esteem among Chinese adolescents: First, the Ministry of Education issued the Guiding Outline of Mental Health Education for Primary and Secondary Schools (2012 revision; Yu et al., 2015). Mental health education and psychological crisis intervention work have been carried out throughout the country. The teaching staff has been strengthened so that mental health education in major schools has embarked on a standardized path. Mental health education and psychological counseling activities in schools can effectively improve the self-esteem of individuals with low self-esteem (Liu et al., 2021). Secondly, due to the increasing comprehensive national strength, the growing investment in education, the gradual improvement of school construction and equipment, and the continuous reform of teaching methods, modern education pays more and more attention to students’ comprehensive quality. Improving students’ performance will also improve self-esteem (Schuster et al., 2013). Third, since the 18th Party Congress, the largest human poverty reduction has been accomplished through precise poverty alleviation, the people’s main status has been effectively reflected and guaranteed, and the economic results have enhanced the people’s national identity. According to social identity theory, the development of individual self-esteem depends to some extent on an individual’s group identity or social identity, and those who have positive evaluations of the group they are in have higher self-esteem than those who have negative evaluations (Whang and Su, 2022). Taken together, the level of self-esteem among Chinese adolescents shows an upward trend.

### Effects of age on the development of self-esteem in adolescents

Previous research on the developmental trajectory of self-esteem has shown that it varies across age groups but generally exhibits a trend of steady growth (Orth et al., 2018). Similarly, the present study found that self-esteem improves as Chinese adolescents grow older.

The present study also revealed another important characteristic of self-esteem development in Chinese adolescents. It was observed that in the first year of each academic period, namely grades 7 (first year) and 10 (first year of high school), there is a decline in self-esteem. Previous research has suggested that self-esteem may decrease during early adolescence due to factors such as heightened social comparisons, reduced individual attention from teachers, and physiological changes associated with puberty (Orth et al., 2018). During puberty, various factors can pose a threat to an individual’s self-esteem, leading to increased self-awareness, self-consciousness, introspection, and self-imagery, which may favor negative self-perceptions. This is a time when adolescents may experience fragile and vulnerable self-esteem and become more cognizant of the relationship between self-esteem, social support, and depressed mood (Harter, 1985). However, in mid-adolescence, individuals’ self-esteem begins to recover, possibly due to increased autonomy, a sense of control, social support, and a better fit between their activities and their personality (Orth et al., 2018).

It is worth noting that a decline also occurs in the 12th grade (senior year) stage. Some studies have proposed that adolescents perceive academic competence, athletic competence, social acceptance, cosmetic appearance, and demeanor (Harter, 1985) as the domains most closely associated with self-esteem. In China, where the college entrance exam is a major life event faced by students and an important means of testing academic level, the higher level of importance and the lower level of ability create inconsistency in self-perception and lower individuals’ self-esteem. As a result, adolescents at this stage experience transient fluctuations in self-esteem.

This implies that schools and families should provide more social support to adolescents during their first year of schooling and senior year of high school. They can do so by offering unconditional praise based on the areas that adolescents perceive as important, such as academic competence and demeanor. This approach can help mitigate the fluctuations in adolescents’ self-esteem during this period.

### Changes and differences in self-esteem levels among different groups of adolescents

Although adolescents’ self-esteem level is increasing year by year, there are differences in the changes among different subgroups.

Firstly, it is noteworthy that self-esteem levels among adolescents of different genders have been increasing over the years, with greater increases observed among girls. However, tests examining the differences in self-esteem between boys and girls show small effect sizes. Previous studies on gender differences have indicated that boys tend to have higher mean self-esteem scores than girls (Orth et al., 2018; Martinez-Escudero et al., 2023). Interestingly, from 1996 to 2019, the level of self-esteem has increased more significantly for girls than for boys.

On one hand, this may be attributed to the fact that girls in adolescence are more sensitive to external evaluations and perceptions (He, 2015). Consequently, as a result of external evaluations and feedback, girls become more aware of and evaluate themselves more clearly, leading to higher levels of self-acceptance. This, in turn, contributes to a greater increase in self-esteem levels for girls compared to boys. On the other hand, this may also indicate that the development of more refined self-esteem content follows a different pattern. Research has shown that females tend to have a higher sense of worth, particularly in the academic and family domains (Reyes et al., 2023; Villarejo et al., 2023). As a result, females are more likely to experience an increase in self-esteem, as evidenced by the higher rise observed. Given that self-esteem encompasses multiple dimensions of evaluation and stems from the integrative evaluation of various self-concepts, the overall difference in self-esteem levels between boys and girls is relatively small. A more nuanced examination of gender differences in self-esteem would require a gender-specific assessment of finer aspects of self-esteem content.

This necessitates that school and family education, in order to effectively enhance the self-esteem levels of both boys and girls, should be tailored and focused on addressing the components of self-esteem that are deemed important by adolescents of different genders. For instance, efforts can be made to increase the level of social approval, enabling adolescents of different genders to develop a sense of competence and value in areas that are individually significant to them.

Furthermore, it is worth noting that the self-esteem of only children has exhibited a higher increase compared to that of non-only children. In the early years, only children had lower levels of self-esteem than non-only children. However, in recent years, only children have demonstrated higher self-esteem levels than their non-only counterparts, resulting in a significant difference in self-esteem between the two groups. Several studies have indicated that only children tend to be less competitive, trusting, and responsible than non-only children. Additionally, only children are often less optimistic and more prone to neuroticism (Cameron et al., 2013).

In the early years, fathers of only children are more likely to adopt negative attitudes in their parenting approach (Zhao et al., 2015). This, coupled with a lower sense of self-identity among only children, contributes to lower self-esteem levels compared to non-only children during this period. Interestingly, current research suggests that only children exhibit higher growth in self-esteem based on the historical evolution of self-esteem. This may be attributed to the fact that previous levels of self-esteem may have been lower or have been improving over time. Some studies have indicated that in recent years, there has been a shift in parents’ educational approach towards their children. Many parents have received higher levels of education, resulting in stronger concepts of equality and democracy. These “Post-80s parents” tend to respect and understand their children, fostering open communication. The increased tolerance and understanding exhibited by parents towards their only child facilitate the child’s self-identification process, leading to a greater increase in their self-esteem levels.

Simultaneously, changes in parenting styles and the relaxation of the one-child policy have resulted in only children receiving more parental attention and care compared to non-only children. Studies have shown that mothers significantly reduce emotional communication with their older children after the birth of younger siblings, whereas parent–child relationships in only-child families tend to be significantly better than in non-only-child families (Baydar and Brooks-Gunn, 1997; Xiao et al., 2017). Parental support happens to be the most effective source of self-esteem development (Liu and Zou, 2007). Due to the dispersion of parental emotional attention in non-only-child families, only children tend to have higher levels of self-esteem compared to non-only children. In previous studies, scholars have primarily focused on the psychological development of only children, neglecting the psychological development of non-only children.

A meta-analysis of parenting styles in Chinese families reveals that only children receive more parental care than non-only children (Zhang, 2011). Currently, China is encouraging the birth of two or three children to further optimize its fertility policy. Therefore, it is crucial to pay attention to the development of self-esteem in non-only children in order to promote the social adaptability of adolescents.

Finally, the self-esteem level of rural adolescents has significantly increased, but the results of urban adolescents are not significant. On the one hand, this may be related to the continuous improvement of China’s comprehensive national strength and the implementation of a series of measures such as targeted poverty alleviation and rural revitalization. The rural population has gradually shaken off poverty and backward living conditions, the living environment has been greatly improved, the gap between rural and urban has narrowed, and rural adolescents can receive a good education. As a result, rural adolescents’ self-esteem has gradually improved. On the other hand, although our country’s urban and rural areas are developing economically, rural and urban areas are still at different stages of economic development. Based on prior research findings (Easterlin and Veenhoven, 2017), short-term rapid economic growth can enhance self-esteem in rural areas with low economic levels. Conversely, for towns with better economic conditions, economic development may not have a significant impact on self-esteem. Finally, literature and sample characteristics can influence a meta-analysis of cross-sectional history (Twenge and Jean, 2001; Twenge et al., 2010b; Xin et al., 2011). There is less literature on the study of self-esteem of rural and urban adolescents. Urbanization construction also shifts rural samples to towns, which may have affected the results.

### The correlation between adolescent self-esteem level and social indicators

The influence of the social environment on individuals is second only to the role of genetics (Twenge and Jean, 2001). However, most current meta-analysis focuses on the individual level or the correlation between age and psychological quantity. Few studies have combined age, social factors, and individuals to analyze the three. As a special meta-analysis method, the cross-sectional history study addresses this problem by explaining the changes in individuals through the changes in social statistics. Therefore, by analyzing the correlation between social indicators and self-esteem, the influence of social factors on adolescent self-esteem can be explained.

The study found that the self-esteem level of adolescents was significantly correlated with the Gini coefficient of the survey year and 5 years ago. Household consumption level, GDP *per capita*, school admission rate, population change, and youth crime rate indicated that adolescent self-esteem changes correlated with demographic characteristics and economic, educational, and social threats. Adolescents are no longer troubled by material needs at the high socio-economic level. They can meet their spiritual needs and continue to explore themselves. At the same time, the stable social environment in our country will make national happiness rise, and the strong traditional culture, sediment, and stable social environment will also imperviously make the teenagers develop in a positive direction. In the process of social development, the self-esteem level of teenagers is also gradually improved.

According to the analysis results, the growth of the economy, the population change, the improvement of education level, and the reduction of social threats all contribute to the improvement of adolescents’ self-esteem levels. Therefore, vigorously developing the economy, improving the quality of education, and ensuring social stability will not only help to improve the living standards of Chinese residents but also have profound significance for the future development of our country. The household consumption level, *per capita* GDP, junior high school enrollment rate, population mortality rate, and juvenile crime rate significantly impact adolescent self-esteem development. This indicates that adolescents are more sensitive to changes in demographic characteristics, economy, education, and social threats and may experience greater social pressure. Society should give adolescents more development opportunities. We should provide adolescents with more attention and support to relieve their stress.

### Research limitations and prospects

There are several limitations to our study. Firstly, the results of this study are based on the use of the SES scale, which is a unidimensional measure of self-esteem. However, current tests of self-esteem employ a multidimensional approach, including measures such as the Domain Variability Measure of Self-Esteem and the Implicit Associations Test for implicit self-esteem. Additionally, more specific measures of self-esteem, such as academic self-esteem and physical self-esteem, could provide a more comprehensive understanding of self-esteem. Therefore, future research could consider incorporating multidimensional self-esteem measures or adopting a longitudinal design to address this limitation.

Furthermore, the results of this study do not establish a causal relationship between social indicators and self-esteem. Causality requires fulfilling three conditions: covariance, temporal order, and the exclusion of other possibilities. In this study, only correlation analyses were conducted, satisfying only one of the covariance conditions. To establish causality, future research could employ quasi-experimental designs or intervention studies.

Additionally, it is important to note that this hypothesis assumes the constancy of the structure of self-esteem among adolescents. However, the structure of adolescents’ self-esteem may change over time. Therefore, future research could explore how the structure of adolescents’ self-esteem evolves in response to societal changes.

## Conclusion

1. Between 1996 and 2019, there was a significant correlation between the overall self-esteem level of Chinese adolescents and chronological age. Chronological age was able to explain 25% of the variance in self-esteem scores, indicating that self-esteem levels tend to gradually increase with age. It is crucial to pay attention to three key time points in the lives of Chinese adolescents and provide strong social support to mitigate any decline in self-esteem.
2. The self-esteem of girls tends to increase more than that of boys, although the gender difference in self-esteem is not significant. This suggests that guidance should be tailored to the specific areas valued by adolescents of different genders. Only children tend to experience a greater increase in self-esteem compared to non-only children, and this difference is significant. Therefore, it is important to focus on addressing self-esteem issues among non-only children when optimizing population reproduction policies. The self-esteem of rural adolescents is significantly correlated with chronological age, while that of urban adolescents is not. The self-esteem level of urban adolescents does not show a correlation with age, whereas the self-esteem level of rural adolescents exhibits a consistent upward trend over the years. This suggests that the self-esteem of rural adolescents has also improved alongside policies aimed at poverty alleviation and rural revitalization, which have contributed to economic advancements.
3. The self-esteem scores of adolescents and their subgroups are significantly correlated with 10 social indicators across four areas: demographic characteristics, economy, education, and social threats. This indicates that social changes can predict changes in adolescents’ self-esteem levels to a certain extent.

## Data availability statement

The datasets presented in this study can be found in online repositories. The names of the repository/repositories and accession number(s) can be found at: https://pan.baidu.com/s/1KD9ICvKWfKnedgkIRUFY1g; Code: j8sc.

## Author contributions

ML: Writing – original draft, Writing – review & editing. QX: Formal analysis, Writing – review & editing. XH: Writing – review & editing, Data curation. YJ: Writing – review & editing, Conceptualization, Funding acquisition. RY: Writing – review & editing, Formal analysis. JL: Supervision, Writing – review & editing.
